# Direct composite veneers for anterio teeth: prevention of asthetic deformation

**DOI:** 10.1186/1878-5085-5-S1-A117

**Published:** 2014-02-11

**Authors:** Bogdan R Shumilovich

**Affiliations:** 1 Voronezh N.N. Burdenko State Medical Academy, Dentistry Department, Voronezh, Russia

## 

Direct restoration of hard dental tissues is the one of the most common method in dentistry. Usually restorations are achieved by hand free technique and a major challenge relates to the management of the anatomical form, size, individual relief and shadows among others. Inadequate due consideration of these aspects can result in deformation of restorations. Coltene/ Whaledent offer solutions to these potential problems.

The system of ready to use composite veneers “Componeer” is a unique product of the Swiss company Coltene/Whaledent; it is the culmination of fifty years of’ experience in the field of composite materials. This system brings together the advantages of direct and indirect methods of restorations. The Componeer is the result of Synergy D6 nanocomposite and offers high mechanical resistance, color stability, and the system of halftone colors which makes the selection of shades easier along with user friendliness. These features allow achievement of great aesthetic results in a very short time. Further, the usage of the original etalon scale gives the ability to estimate the optical qualities of hard dental tissues and to develop the strategy of the restoration at the outset. Creation of the Componeer matrix, polymerization and polishing, at the workshop conditions, gives the dental professional the ability to optimize the effects of the fluorescence, opalescence and “chameleon” in full.

In conclusion, the “Componeer” has emerged as a serious alternative to indirect restorations with the ability to achieve clinically effective restorations with high aesthetic quality.

